# A Tractable Method for Describing Complex Couplings between Neurons and Population Rate

**DOI:** 10.1523/ENEURO.0160-15.2016

**Published:** 2016-08-18

**Authors:** Christophe Gardella, Olivier Marre, Thierry Mora

**Affiliations:** 1 Laboratoire de Physique Statistique, Centre National de la Recherche Scientifique, École Normale Supérieure, Université Pierre et Marie Curie, 75005 Paris, France; 2 Institut de la Vision, Institut National de la Santé et de la Recherche Médicale, Université Pierre et Marie Curie, 75012 Paris, France

**Keywords:** maximum entropy models, population couplings, retinal ganglion cells, tuning curve

## Abstract

Neurons within a population are strongly correlated, but how to simply capture these correlations is still a matter of debate. Recent studies have shown that the activity of each cell is influenced by the population rate, defined as the summed activity of all neurons in the population. However, an explicit, tractable model for these interactions is still lacking. Here we build a probabilistic model of population activity that reproduces the firing rate of each cell, the distribution of the population rate, and the linear coupling between them. This model is tractable, meaning that its parameters can be learned in a few seconds on a standard computer even for large population recordings. We inferred our model for a population of 160 neurons in the salamander retina. In this population, single-cell firing rates depended in unexpected ways on the population rate. In particular, some cells had a preferred population rate at which they were most likely to fire. These complex dependencies could not be explained by a linear coupling between the cell and the population rate. We designed a more general, still tractable model that could fully account for these nonlinear dependencies. We thus provide a simple and computationally tractable way to learn models that reproduce the dependence of each neuron on the population rate.

## Significance Statement

The description of the correlated activity of large populations of neurons is essential to understand how the brain performs computations and encodes sensory information. These correlations can manifest themselves in the coupling of single cells to the total firing rate of the surrounding population, as was recently demonstrated in the visual cortex, but how to build this dependence into an explicit model of the population activity is an open question. Here we introduce a general and tractable model based on the principle of maximum entropy to describe this population coupling. By applying our approach to multielectrode recordings of retinal ganglion cells, we find complex forms of coupling, with the unexpected tuning of many neurons to a preferred population rate.

## Introduction

An important feature of neural population codes is the correlated firing of neurons. Manifestations of collective activity are observed in the correlated firing of individual pairs of neurons (Arnett, 1978), and through the coupling of single neurons to the activity in its surrounding population (Arieli et al., 1996; Tsodyks et al., 1999). These correlations, whether they are evoked by common inputs or result from interactions between neurons, imply that the neural code must be studied through the collective patterns of activity rather than by individual neuron.

As the number of possible firing patterns in a population grows exponentially with its size, they cannot be sampled exhaustively for large populations. Several modeling approaches have been suggested to describe the collective activity patterns of a neural population (Martignon et al., 1995; Schneidman et al., 2003, 2006; Pillow et al., 2008; Cocco et al., 2009; Tkačik et al., 2014). In these approaches, a small number of statistics (e.g., mean firing rate, pairwise correlations) is measured to constrain the parameters of the model. Models are then evaluated on their ability to predict statistics of the population activity that were not fitted to the data. These models are computationally hard to infer, and one must usually have recourse to approximate methods to fit them.

Recently, Okun et al. (2015) investigated how the activity of the whole population influenced the behavior of single neurons in the primary visual cortex of awake mice and monkeys. In particular, they studied the role of the correlation between neurons and the summed activity of the population, called the population rate. To assess whether these couplings between neurons and population activity were sufficient to describe the correlative structure of the code, synthetic spike trains preserving these couplings were generated and compared to data. However, the numerical method used to generate synthetic spike trains is computationally heavy, and is unable to predict the probability of particular patterns of spikes, as most of them are unlikely to ever occur.

Here we introduce a new method, based on the principle of maximum entropy, to exactly account for the coupling between individual neurons and the population rate. This model is tractable, meaning that predictions for the statistics of the activity can be derived analytically. The gradient and Hessian of the likelihood of the model can thus also be computed efficiently, allowing for fast inference using Newton’s method. Compared with previous methods (Okun et al., 2015), our method can fit hours of large-scale recordings of large populations in a few seconds on a standard laptop computer. We tested it on recordings of the salamander retina (160 neurons). We uncovered new ways for individual neurons to be coupled to the population, where a single neuron is tuned to a particular value of the population rate, rather than being monotonically coupled to the population.

## Materials and Methods

### Recordings from retinal ganglion cells

We analyzed previously published *ex vivo* recordings from retinal ganglion cells of the tiger salamander (*Ambystoma tigrinum*; Tkačik et al., 2014). In brief, animals were killed according to institutional animal care standards. The retina was extracted from the animal, maintained in an oxygenated Ringer’s solution, and recorded on the ganglion cell side with a 252-electrode array. Spike sorting was performed with custom software (Marre et al., 2012), and *N* = 160 neurons were selected for the stability of their spike waveforms and firing rates, and the lack of refractory period violation.

### Maximum entropy models

**Figure 1.**
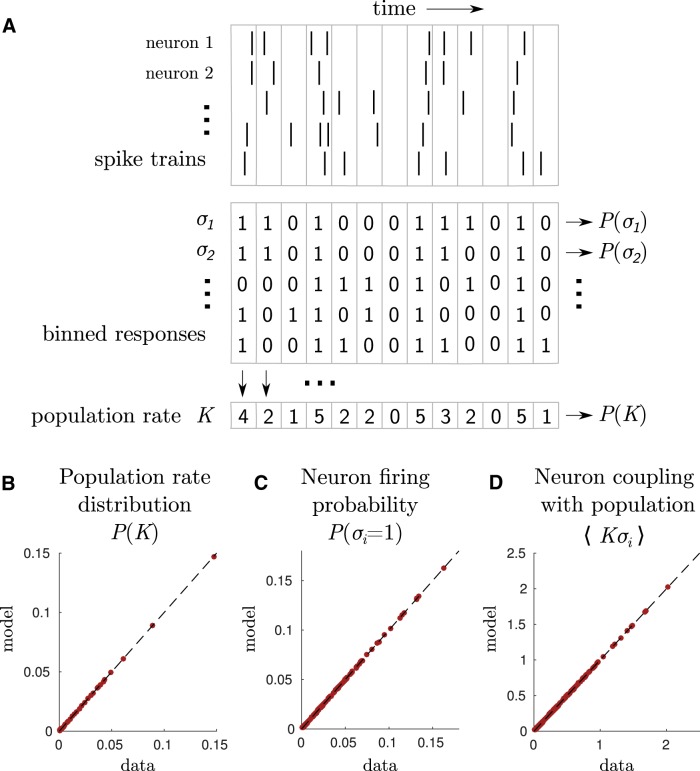
A maximum entropy model for population coupling. ***A***, Spikes trains are recorded with a multielectrode array and binned in 20 ms time windows. We study the dependence between the binned response of each neuron $\sigma_{i}$ and the population rate *K*, defined as the summed activity of all neurons. ***B–D***, The linear-coupling model fits the following three observables with high accuracy: the population rate distribution (***B***); the cells firing rates (***C***); and population couplings (***D***). For each observable, the model fit is plotted against empirical values.

We are interested in modeling the probability distribution of population responses in the retina. The responses are first binned into 20 ms time intervals. The response of neuron *i* in a given interval is represented by a binary variable, $\sigma_{i}$, which takes value 1 if the neuron spikes in this interval, and 0 if it is silent. The population response in this interval is represented by the vector $\sigma =(\sigma_{1},\ldots ,\sigma_{N})$ of all neuron responses (Fig. 1*A*). We define the population rate *K* as the number of neurons spiking in the interval $K(\sigma )=\overset{N}{\underset{i=1}{\sum }}\sigma_{i}$.

We build three models for the probability of responses P(σ). These models reproduce some chosen statistics, meaning that these statistics have same value in the model and in empirical data. The first model reproduces the firing rate of each neuron and the distribution of the population rate. The second model also reproduces the correlation between each neuron and the population rate. The third model reproduces the whole joint probability of single neurons with the population rate. It is a hierarchy of models, because the statistics of each model are also captured by the next one.

#### Minimal model

We build a first model that reproduces the firing rate of each neuron, $P(\sigma_{i}=1)=〈\sigma_{i}〉$, and the distribution of the population rate, *P*(*K*). We also want the model to have no additional constraints, and thus be as random as possible. In statistical physics and information theory, the randomness of a distribution *P* is measured by its entropy *S*(*P*):

(1) $$S(P)=-\underset{\sigma }{\sum }P(\sigma )\;\ln\;P(\sigma ),$$

where the sum runs over all possible states. The maximum entropy model is the distribution that maximizes this entropy while reproducing the constrained statistics. Using the technique of Lagrange multipliers (see Mathematical derivations), one shows that the model must take the following form:

(2) $$P(\sigma )=\frac{1}{Z}\exp\left(\overset{N}{\underset{i=1}{\sum }}\left(\alpha_{i}+\beta_{K(\sigma )}\right)\sigma_{i}\right),$$

where the parameters *α_i_*, *i* = 1,…,*N* and *β_K_*, *K* = 0,…,*N* must be fitted so that the distribution of Equation 2 matches the statistics $〈\sigma_{i}〉$ and *P*(*K*) of the data. *Z* is a normalization factor. Note that $\beta_{K(\sigma )}$ depends on the state σ through K(σ). We refer to this distribution as the minimal model, as no explicit dependency between the activity of individual neurons and the population rate is constrained.

#### Linear-coupling model

The second model reproduces $〈\sigma_{i}〉$ and *P*(*K*) as before, as well as the linear correlation $〈K\cdot \sigma_{i}〉$ between each neuron response $\sigma_{i}$ and the population rate *K*, for *i* = 1,…,*N*. It takes the following form (see Mathematical derivations):

(3) $$P(\sigma )=\frac{1}{Z}\exp\left(\overset{N}{\underset{i=1}{\sum }}\left(\alpha_{i}+\beta_{K(\sigma )}+\gamma_{i}K\right)\sigma_{i}\right).$$

Analogously to the minimal model, the parameters *α_i_*, *β_K_*, and *γ_i_* are inferred so that the model agrees with the mean statistics $〈\sigma_{i}〉$, *P*(*K*), and $〈K\cdot \sigma_{i}〉$ of the data. Importantly, despite their common notation, the values of the fitted parameters *α_i_* and *β_K_* are different from the ones fitted in the minimal model (see Mathematical derivations).

#### Complete-coupling model

The third model reproduces the joint probability distributions of the response of each neuron and the population rate $P(\sigma_{i},K)$. It takes the following form (see Mathematical derivations):

(4) $$P(\sigma )=\frac{1}{Z}\exp\left(\sum_{i=1}^{N}h_{iK(\sigma )}\sigma_{i}\right).$$

The parameters ${\left(h_{iK}\right)}_{i=1,\ldots ,N;K=0,\ldots ,N}$ are inferred so that the model agrees with the data on $P(\sigma_{i},K)$ for each (*i*, *K*) pair. Note that $h_{iK(\sigma )}$ depends on the state σ. We refer to this model as the complete-coupling model since it reproduces exactly the joint probability between each neuron and the population rate.

### Model solution

The minimal and linear-coupling models can be written in the same form as the complete-coupling model (Eq. 4), but with constraints on the form of *h_iK_*. In the minimal model, the matrix *h_iK_* is constrained to have the form $\alpha_{i}+\beta_{K}$. In the linear-coupling model, it is constrained to have the form *α_i_* + *β_K_* + *γ_i_ K*. In the complete-coupling model, the matrix *h_iK_* has no imposed structure, and all its elements must be learned from the data.

Since all the considered models can be viewed as subcases of the complete-coupling model (Eq. 4), we only describe the mathematical solution to this general case. First, we describe how to solve the direct problem, i.e., how to compute statistics of interest, such as *P*(*σ_i_*,*K*), from the parameters *h_iK_*. In the next section, we explain how to solve the inverse problem—the reverse task of inferring the model parameters from the statistics—which relies on the solution to the direct problem.

A model is considered tractable if there exists an analytical expression for the normalization factor,

(5) $$Z=\underset{\sigma }{\sum }\exp\left(\overset{N}{\underset{i=1}{\sum }}h_{iK(\sigma )}\sigma_{i}\right),$$

allowing for its rapid computation (e.g. in polynomial time in *N*). All statistics of the model, such as *P*(*σ_i_*,*K*) or covariances $〈\sigma_{i}\sigma_{j}〉-〈\sigma_{i}〉〈\sigma_{j}〉$ between pairs of neurons, can then be calculated efficiently through derivatives of *Z* (see Mathematical derivations). In general, maximum entropy models are not tractable, because sums of the kind in Equation 5 involve a sum over an exponential number of terms (2*^N^*). Fortunately, in our case, the technique of probability-generating functions provides an expression for *Z* that is amenable to fast computation using polynomial algebra (see Mathematical derivations), as follows:

(6) $$Z=\overset{N}{\underset{K=0}{\sum }}\text{Coeff}\left[\overset{N}{\underset{i=1}{\prod }}\left(1+Xe^{h_{iK}}\right),X^{K}\right],$$

where $\text{Coeff}[Q,X^{n}]$ denotes the coefficient of polynomial *Q* of order *X^n^*.

### Model inference

We now describe how to fit the models to experimental data. The inference of the model parameters is equivalent to a problem of likelihood maximization (Ackley et al., 1988). The model reproduces the empirical statistics exactly when the parameters maximize the likelihood of experimental data measured by the model, $L=\overset{n}{\underset{\alpha =1}{\prod }}P(\sigma^{\left(\alpha \right)})$, where $\left(\sigma^{\left(1\right)},\ldots ,\sigma^{\left(n\right)}\right)$ are the *n* activity patterns recorded in the experiment, assumed to be independently drawn.

In practice, we maximized the normalized log-likelihood L=(1/n)log L instead of *L*, which is equivalent theoretically but is more convenient for computation. We used Newton’s method to perform the maximization. This method requires computation of the first and second derivatives of the normalized log-likelihood. These derivatives can be expressed as functions of mean statistics of the model and can be calculated using the solution to the direct problem sketched in the previous section, and detailed in the Mathematical derivations section. Because the model is tractable, these mean statistics can be computed quickly, and the model can be inferred rapidly.

For the minimal model, the optimization was performed over the parameters ${\left(\alpha_{i}\right)}_{i=1,\ldots ,N}$ and ${\left(\beta_{K}\right)}_{K=0,\ldots ,N}$. For the linear-coupling model, the optimization was performed over these two sets of parameters, as well as ${\left(\gamma_{i}\right)}_{i=1,\ldots ,N}$. For the complete-coupling model, the optimization was performed over all elements of the matrix ${\left(h_{iK}\right)}_{i=1,\ldots ,N;K=0,\ldots ,N}$. We stopped the algorithm when the fitting error was smaller than 10^−6^ (see Mathematical derivations).

### Regularization

Prior to learning the model, we regularized the empirical population rate distribution *P*(*K*) and conditional firing rates $P(\sigma_{i}|K)$ to mitigate the effects of low sampling noise. This regularization allowed us to remove zeros from the mean statistics, avoiding issues with the fitting procedure. We performed this regularization using pseudocounts (see Mathematical derivations).

### Tuning curves in the population rate

We define the tuning curve of neuron *i* in the population rate as the conditional probability of neuron *i* to spike given the summed activity of all neurons but *i*, $K_{\backslash i}=\underset{j\neq i}{\sum }\sigma_{j}$. It is equal to the following:

(7) $$p(\sigma_{i}=1|K_{\backslash i})=\frac{P(\sigma_{i}=1,K_{\backslash i})}{P(\sigma_{i}=0,K_{\backslash i})+P(\sigma_{i}=1,K_{\backslash i})},$$

where we can use $P(\sigma_{i}=1,\;K_{\backslash i})=P(\sigma_{i}=1,\;K=K_{\backslash i}+1)$ and $P(\sigma_{i}=0,\;K_{\backslash i})=P(\sigma_{i}=1,\;{K=K}_{\backslash i})$. Each of these quantities can be computed using the solution to the direct problem (see Mathematical derivations).

We then tested for each neuron whether its tuning curve had significant local maxima. We first identified the set of $K_{\backslash i}$ for which $P(\sigma_{i}=1|K_{\backslash i})$ was significantly larger than points below and above $K_{\backslash i}$. To assess significance, we measured the SD of the difference across 100 training sets consisting of random halves of the dataset. The difference was said to be significant when it was 5 SDs above 0.

For the cells for which the presence of a maximum was determined, we then evaluated the location of the maximum, $K_{\backslash i}^{*}$, by taking the median of the maxima determined for each training set. We inferred the presence and position of minima in a similar way.

To estimate the quality of the model prediction for the tuning curve, we quantified how the model differed from the data. We trained the model on 100 random training sets and computed $D_{KL}(\text{test}\|\text{model})$, the difference between $P(\sigma_{i},K)$ in the testing data and predicted by the model, measured by the Kullback–Leibler (KL) divergence. The KL divergence between two distributions *P* and *Q* of a random variable *x* is: $D_{KL}(P\|Q)=\sum_{x}P(x)\text{log}[P(x)/Q(x)]$. We regularized $P(\sigma_{i},K)$ in the testing set before computing the KL divergence. To measure sampling noise, we computed the difference between the testing and the training sets, *D_KL_*(test ‖ train), where $P(\sigma_{i},K)$ was regularized in both sets. The normalized KL divergence, *z*, is defined as the difference between *D_KL_* (test ‖ model) and *D_KL_* (test ‖ train), divided by the SD, as follows:

(8) $$z=\frac{\text{mean}\left(D_{KL}(\text{test}\|\text{model})-D_{KL}(\text{test}\|\text{train})\right)}{\text{std}\left(D_{KL}(\text{test}\|\text{model})-D_{KL}(\text{test}\|\text{train})\right)}.$$

In other words, it measures by how many SDs the data differ from the model.

### Quality of the model

#### Pairwise correlations

In order to measure the quality of the predictions of correlations between pairs of neurons $\sigma_{i}$ and $\sigma_{j}$, we used cross-validation. We randomly divided the dataset into 100 training and testing sets half the size of the data, and learned the model on the training sets. The correlations of each testing set $c_{\text{test},ij}$ were then predicted with the model $c_{\text{model},ij}$. The quality of the model prediction was measured by a goodness-of-fit index quantifying the amount of correlations predicted by the model. We define it as follows:

(9) $$C=\frac{\underset{i<j}{\sum }c_{\text{test},ij}^{2}-\underset{i<j}{\sum }{\left(c_{\text{test},ij}-c_{\text{model},ij}\right)}^{2}}{\underset{i<j}{\sum }c_{\text{test},ij}^{2}-\underset{i<j}{\sum }{\left(c_{\text{test},ij}-c_{\text{train},ij}\right)}^{2}},$$

where $c_{\text{train},ij}$ is the correlation in the corresponding training set. The numerator of Equation 9 is the part of the correlations in the testing set that is predicted by the model, and the lower one is a normalization correcting for sampling noise. We have *C* = 1 when the model perfectly accounts for the correlations of the training set. When the model completely ignores correlations, as in a model of independent neurons, *c_ij_* = 0, then *C* = 0.

#### Likelihood

Using the models learned on the same 100 training sets, we computed the likelihood of responses in the testing sets for the minimal, linear-coupling, and complete-coupling models. In this article, the log-likelihood is expressed in bits, using binary logarithms. We then computed the improvement in mean log-likelihood compared to the minimal model, for complete versus linear models as the ratio ${〈{〈\log P_{\text{complete}}(\sigma )-\log P_{\text{minimal}}(\sigma )〉}_{\sigma }/{〈\log P_{\text{linear}}(\sigma )-\log P_{\text{minimal}}(\sigma )〉}_{\sigma }〉}_{\text{test}}$, where ${〈\cdot 〉}_{\text{test}}$ is the mean over training sets and ${〈\cdot 〉}_{\text{σ}}$ is the mean over responses in each testing set.

#### Multi-information

The multi-information (Cover and Thomas 1991; Schneidman et al., 2003) quantifies the amount of correlative structure captured by a model. It is defined as the difference between the entropy of a model of independent neurons reproducing firing rates and the empirical data: *I* = *S*_indep_ − *S*_data._ Here $S_{\text{data}}=-\underset{\sigma }{\sum }P_{\text{data}}(\sigma )\log P_{\text{data}}(\sigma )$ is the entropy of the spike patterns measured by their frequencies, $P_{\text{data}}(\sigma )$, in the data, and *S*_indep_ is the entropy if all neurons were independent.

The entropy of a maximum entropy model is by construction higher than that of the real data, $S_{\text{model}}>S_{\text{data}}$, because the model has maximum entropy given the statistics it reproduces. Its entropy is also smaller than *S*_indep_, provided that constraints include the spike rates, because the model has more structure and reproduces more statistics than if neurons were independent. Thus, the fraction of correlations that is accounted by the maximum entropy model, $0<I_{\text{model}}/I<1$, where $I_{\text{model}}=S_{\text{indep}}-S_{\text{model}}$, can be viewed as a measure of how well the model captures the correlative structure of responses. The true multi-information *I* can only be calculated for small groups of neurons (N≤20), because *P*_data_ requires evaluation of 2*^N^* pattern frequencies, which is prohibitive for large networks.

## Results

### Tractable maximum entropy model for coupling neuron firing to population activity

The principle of maximum entropy (Jaynes, 1957a,b) provides a powerful tool to explicitly construct probability distributions that reproduce key statistics of the data, but are otherwise as random as possible. We introduce a novel family of maximum entropy models of spike patterns that preserve the firing rate of each neuron, the distribution of the population rate, and the correlation between each neuron and the population rate, with no additional assumptions (Fig. 1*A*). Under these constraints, the maximum entropy distribution over spike patterns in a fixed 20 ms time window is given by the following (see Materials and Methods):

(10) $$P(\sigma_{1},\ldots ,\sigma_{N})=\frac{1}{Z}\exp(\overset{N}{\underset{i=1}{\sum }}\left(\alpha_{i}+\beta_{K}+\gamma_{i}K\right)\;\sigma_{i}),$$

where $\sigma_{i}$ equals 1 when neuron *i* spikes within the time window, and 0 otherwise, $K=\sum_{i}\sigma_{i}$ is the population rate, and *Z* is a normalization constant. The parameters ${\left(\alpha_{i}\right)}_{i=1,\ldots ,N},\;{\left(\gamma_{i}\right)}_{i=1,\ldots ,N}$ and ${\left(\beta_{K}\right)}_{K=0,\ldots ,N}$ must be fitted to empirical data. We refer to this model as the linear-coupling model, because of the linear term $\gamma_{i}K\sigma_{i}$ in the exponential.

Unlike maximum entropy models in general, this model is tractable, meaning that its prediction for the statistics of spike patterns has an analytical expression that can be computed efficiently using polynomial algebra. This allows us to infer the model parameters rapidly for large populations on a standard computer, using Newton’s method (see Materials and Methods). We learned this model in the case of a population of *N* = 160 salamander retinal ganglion cells, stimulated by a natural movie. It took our algorithm 14 s to fit the 3*N*–2 model parameters (see Mathematical derivations) so that the maximum discrepancy between the model and the data was <10^−6^ (Fig. 1*B–D*).

The linear-coupling model provides a rigorous mathematical formulation for the hypotheses underlying the modeling approach of Okun et al. (2015) applied to cortical populations. In that work, synthetic spike trains were generated by shuffling spikes from the original data so as to match the three constraints listed above on the single-neuron spike rates, the distribution of population rates, and their linear correlation. Shuffling data (i.e., increasing randomness and hence entropy while constraining mean statistics) have previously been shown to be equivalent to the principle of maximum entropy in the context of pairwise correlations (Bialek and Ranganathan, 2007). Our formulation provides a fast way to learn the model and to make predictions from it, as we shall see below. In addition, it allows us to calculate the probability of individual spike patterns (Eq. 10), which a generative procedure such as the one in Okun et al. (2015) cannot.

### Tuning curves of single neurons to the population activity

**Figure 2.**
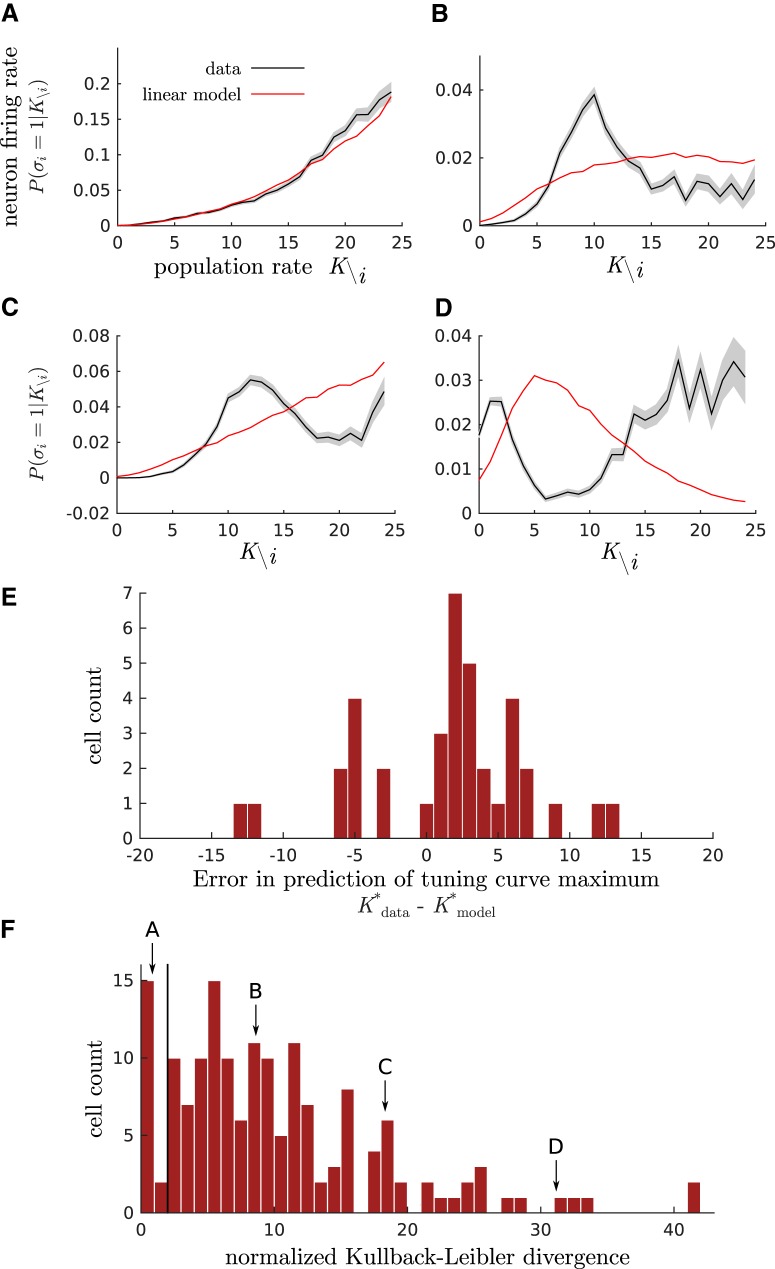
Tuning curves of single neurons as a function of the population rate. ***A–D***, Spiking probability of neuron *i* conditioned on the summed activity of all other neurons $P(\sigma_{i}=1|K_{\backslash i})$ , as observed in the data (black curves; SE is shaded in gray) and predicted by linear-coupling model (red curves). Each subfigure corresponds to a different representative cell. ***E***, Histogram of the difference between the preferred population rate—at which the tuning curve is maximal—observed in the data, $K_{\text{data}}^{*}$ , and predicted by the linear-coupling model, $K_{\text{model}}^{*}$ . Data are shown for the 38 cells that had at least one local maximum both in the linear model and in the data. When the empirical tuning curve had two local maxima, the closest one to the model prediction was chosen. ***F***, Histogram of the normalized KL divergence between the observed joint distributions $P(\sigma_{i},K)$ and its prediction by linear-coupling model. The arrows indicate the value for the four example cells ***A–D***. The vertical line shows a normalized divergence of 2, meaning that cells sitting on its right deviate from the linear-coupling model by >2 SDs.

We wondered whether the linear-coupling model could explain how the response of single neurons depended on the population rate. We examined the firing probability of neuron *i* as a function of the summed activity of the other neurons $K_{\backslash i}=\sum_{j\neq i}\sigma_{j}$, denoted by $P\left(\sigma_{i}=1|K_{\backslash i}\right)$. This quantity can be viewed as the tuning curve of neuron *i* in response to the rest of the population. It can be calculated analytically from the parameters of the model (see Materials and Methods) and compared with empirical values. The tuning curves of four representative cells are shown in Figure 2*A–D*.

The linear-coupling model predicts a variety of tuning curves (in red), from sublinear to superlinear. Although its prediction was qualitatively close to the empirical value for some cells (Fig. 2*A*), the model generally did not account well for the coupling between $\sigma_{i}$ and $K_{\backslash i}$. A majority of cells (85 of 160) displayed a local maximum in their empirical tuning curves, at some preferred value $K_{\backslash i}^{*}$ of the population activity to which the neuron is tuned. The model did not predict the existence of this maximum in 47 of these 85 cells (Fig. 2*C*). Even when it did, the location of the maximum, $K_{\backslash i}^{*}$, was poorly predicted, as can be seen by the distribution of the difference between the model and the data (Fig. 2*E*). In six cases, the tuning curve had two local maxima, while the model only predicted one. Another 27 cells had a minimum in their empirical tuning curve, which was never reproduced by the model (Fig. 2*D*). Interestingly, no cells were tuned to fire when the rest of the population is silent; even cells whose spiking activity was anticorrelated with the rest of the population had a nonzero preferred population rate, $K_{\backslash i}^{*}>0$.

The model performance can be quantified by computing the KL divergence between the data and the model for the joint probability $P(\sigma_{i},K)$ of the neuron and population activity (Fig. 2*F*). The KL divergence is a measure of the dissimilarity between two distributions, *P* and *Q* (Cover and Thomas, 1991), which quantifies the amount of information that is lost if we use *Q* to approximate *P*. We calculated a normalized KL divergence (see Materials and Methods) measuring by how many SDs the KL divergence between the linear-coupling model and the data deviated from what would be expected from sampling noise (Fig. 2*F*). A majority of cells (143 of 160) deviated by >2 SDs, meaning that their tuning curve was not well accounted for by the linear-coupling model. This observation is consistent with the failure of the model to account for the qualitative properties of their tuning curves.

Together, these results indicate that the full dependency between single cells and the population rate cannot be explained by their linear correlation only.

### A refined maximum entropy model

To overcome the limitations of the linear-coupling model, and to fully account for the variety of tuning curves found in data, we built a maximum entropy model constrained to match all joint probabilities of the population rate with each single neuron response, $P(\sigma_{i},K)$. This model takes the following form (see Materials and Methods):

(11) $$P(\sigma_{1},\ldots ,\sigma_{N})=\frac{1}{Z}\exp\left(\sum_{i=1}^{N}h_{iK}\sigma_{i}\right),$$

where the parameters *h_iK_* for *i* = 1,…,*N* and *K* = 0,…,*N* are fitted to empirical data, and *Z* is a normalization constant. Note that the linear-coupling model can be viewed as a particular case of this model, with parameters *h_iK_* constrained to take the form $h_{iK}=\alpha_{i}+\beta_{K}+\gamma_{i}K$. By construction, this model exactly reproduces the tuning curves of Figure 2*A–D*.

Although this model has many more parameters than the simpler linear-coupling model, it is still tractable, and we could infer its *N*(*N* – 1) + 1 parameters (see Mathematical derivations) in 7 s for the whole population of 160 neurons. Hereafter, we refer to this model as the complete-coupling model.

### Pairwise correlations

**Figure 3.**
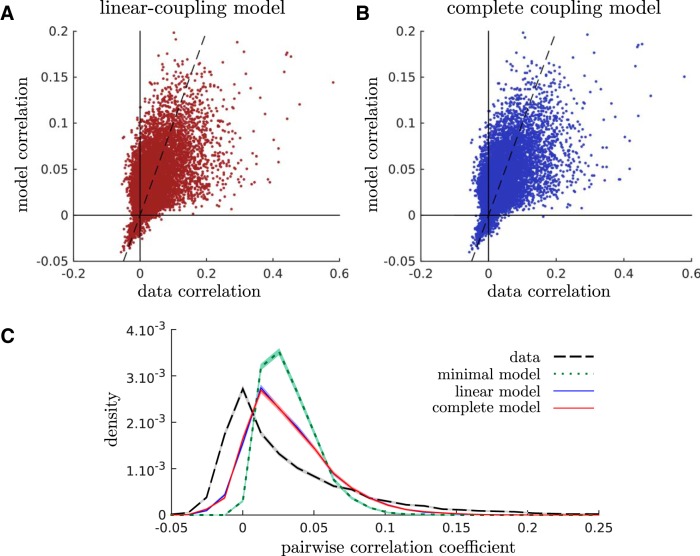
Maximum entropy models of population coupling partly account for pairwise correlations. ***A***, ***B***, The observed correlation coefficient between all pairs of neurons is compared with its prediction according to the linear-coupling (***A***) and complete-coupling (***B***) models. ***C***, Distribution of pairwise correlation coefficients, as observed in the data and predicted by minimal, linear-coupling, and complete-coupling models.

The models introduced thus far are only constrained to reproduce the firing rate of each neuron, the distribution of population rates, and the coupling of each neuron with the population rate. We asked whether these simple models could account for correlations between individual pairs of cells, which were not fitted to the data. The correlation between two neurons, $〈\sigma_{i}\sigma_{j}〉-〈\sigma_{i}〉〈\sigma_{j}〉$, can be calculated analytically from the model parameters (see Materials and Methods) and directly compared with the data (Fig. 3*A*,*B*).

In order to understand the importance of the population rate coupling for the prediction of pairwise correlations, we built a null model constrained only by the firing rates of each neuron and the distribution of the population rate. This simpler maximum entropy model reads as follows:

(12) $$P(\sigma_{1},\ldots ,\sigma_{N})=\frac{1}{Z}\exp\left(\sum_{i=1}^{N}\left(\alpha_{i}+\beta_{K}\right)\;\sigma_{i}\right).$$

We call it the minimal model. Interactions between neurons derive only from the fluctuations of the population activity, rather than from an explicit coupling. This model has 2*N* − 1 parameters (see Mathematical derivations), which are inferred using the same techniques as before.

To quantify the performance of the different models, we calculated a goodness-of-fit index ranging from 0, when the correlations were not predicted at all, to 1, when they were predicted perfectly (see Materials and Methods). This index was 0.380 ± 0.001 for the minimal model, 0.526 ± 0.002 for the linear-coupling model, and 0.544 ± 0.002 for the complete-coupling model. Thus, a substantial amount of pairwise correlations could be explained from the coupling of neurons to the population. By this measure, the complete model performed slightly (but significantly) better than the linear-coupling model.

Figure 3*C* shows the distribution of the pairwise correlations in the data and as predicted by the three models. The minimal model fails to reproduce the long tail of large correlations, and predicts no negative correlations, while 28% of empirical correlations are negative. By contrast, the linear-coupling and complete-coupling models predict 7.6% and 7.7% of negative interactions, respectively, and a longer tail of large correlation coefficients. Thus, the coupling to the population rate is important to reproduce both large correlations and the strong asymmetry of the distribution.

### Prediction of probabilities of spike patterns

We quantified the capacity of models to describe population responses by computing the probability of responses predicted by each model. The mean log-likelihood of responses was −33.10 ± 0.06 bits for the minimal model, −30.12 ± 0.06 bits for the linear-coupling model, and −29.49 ± 0.06 bits for the complete-coupling model. The improvement in mean log-likelihood compared with the minimal model was 51.3 ± 0.5% higher for the complete-coupling model than for the linear-coupling model, meaning that nonlinear couplings to the population are important to model the probability of responses.

The multi-information I (Cover and Thomas, 1991) quantifies, in bits, the amount of correlations in the response, whether they are pairwise or of higher order (see Materials and Methods). To assess the performance of the models in capturing the collective behavior of the networks, we calculated the ratio of the multi-information explained by the model to that estimated directly from the data, *I*_model_ /*I*_data._ This ratio gives a measure of how well the probability of particular spike patterns is predicted by the model: it is 1 when the model is a perfect description of the data, and 0 when the model assumes independent neurons with no correlation between them. Because it requires the estimation of the probability of all possible spike patterns of the populations, the multi-information can be only calculated for small populations of, at most, 20 cells.

With this measure, the linear coupling model could account for 65% of the multi-information for groups of 10 neurons, and 53% for groups of 20 neurons. The complete model slightly improved these ratios to 68% and 56%, respectively (Table 1). Thus, more than half of the correlative structure in the spike patterns could be explained by the coupling to the population rate alone.

**Table 1:** Multi-information *I* estimated either directly from the data or from maximum entropy models, for random subpopulations of 10 and 20 neurons (100 subpopulations each), as well as the ratio of the multi-information between model and data

		Data	Minimal	Linear	Complete
*N* = 10	*I*	0.0713 ± 0.0398	0.0343 ± 0.0268	0.0478 ± 0.0315	0.0498 ± 0.0326
	$I/I_{\text{data}}$	1	0.444 ± 0.135	0.649 ± 0.108	0.677 ± 0.108
*N* = 20	*I*	0.3188 ± 0.0967	0.1291 ± 0.0558	0.1702 ± 0.0592	0.1789 ± 0.0606
	$I/I_{\text{data}}$	1	0.393 ± 0.071	0.531 ± 0.055	0.557 ± 0.055

Results are reported as the Mean (± SD) in bits.

## Discussion

In this study, we have introduced a general computational model for coupling individual neurons to the population rate. This model formalizes and simplifies the generative procedure proposed by Okun et al. (2015) to study population coupling, and overcomes its computational difficulties. In addition, it allows for nonlinear coupling to the population rate.

We have used our model to investigate population coupling in large recordings of *N* = 160 retinal ganglion cells. We found that most cells had a nonlinear coupling to the population rate. In particular, a large fraction of cells were tuned to a preferred value of the population rate. Even more strikingly, a few cells had a least preferred population rate (i.e., they were more likely to spike at lower or higher populations rates). We found no cell that was maximally active when all other neurons were silent, even among cells that were anticorrelated with the population rate. These results emphasize the need for the nonlinear coupling afforded by our model, as they uncover new dependencies that do not fit within the proposed division between soloists and choristers (Okun et al., 2015), such as the tuning to a specific population rate. It would be interesting to test whether these nonlinear couplings can also be found at the cortical level.

Overall, our model reaches a similar predictive performance than what was found in the cortex. The coupling to the population rate accounted for more than half of the correlations between pairs of neurons. In Okun et al. (2015), a custom measure of the fraction of explained pairwise correlations (different from the one used in the present work) gave 0.34. Applying the same measure to our case yields a similar value (0.33). However, this similarity in performance can be due to different underlying mechanisms. In the retina, most correlations are due to common input from previous layers (Trong and Rieke, 2008), while ganglion cells do not make synaptic connections to each other. In contrast, at the cortical level, a larger part of the variability in the activity should be due to internal dynamics generated by recurrent connections (Arieli et al., 1996; van Vreeswijk and Sompolinsky, 1996; Tsodyks et al., 1999). It would be interesting to test our model on cortical data to see whether these differences result in different types of nonlinear population coupling.

Our maximum entropy model of population coupling is complementary to maximum entropy models reproducing correlations between all pairs of neurons. Pairwise models have been shown to accurately describe the collective activity of retinal ganglion cells (Schneidman et al., 2006; Shlens et al., 2006, 2009; Ganmor et al., 2011a,b; Tkačik et al., 2014), and in cortical networks *in vitro* (Tang et al., 2008) and *in vivo* (Yu et al., 2008), but they are not tractable, requiring summation over all 2^N^ possible spiking states in order to implement Boltzmann machine learning (Ackley et al., 1988). Alternative methods based on mean field approximations (Cocco et al., 2009; Cocco and Monasson, 2011) or Monte-Carlo simulations (Broderick et al., 2007) have been proposed. However, Monte-Carlo methods require hours of computations, although recent efforts have tried to lower these computation times for moderately large populations (Ferrari, 2015).

By contrast, the models of population couplings introduced here are much easier to solve. They are tractable, so their predictions can be computed analytically in time *N* ^3^, and their parameters can be inferred in a few seconds on a personal computer from large-scale, hour-long recordings of spike trains for a population of *N* = 160 neurons. These models can then be used to generate synthetic spike trains to calculate analytically response statistics, such as pairwise correlations, or to estimate the probability of particular spike trains. Compared with the shuffling method described by Okun et al. (2015), which is equivalent to the linear-coupling model, our method is simpler and computationally less intensive.

The procedure is general and can be applied to any multineuron recording of individual spikes. The speed of model inference could prove to be an important advantage when studying very large populations, which can now reach 1000 cells (Schwarz et al., 2014). In the case of the linear-coupling model, the number of parameters is also smaller, scaling with the population size *N* rather than *N* ^2^ for the pairwise correlations model.

Note that the population coupling models introduced here belong to a different class than the pairwise models. Each class captures the features of neural responses that the other cannot: models of population coupling should be sufficient for studying the global properties of collective activity, while pairwise models are still needed to account for the detailed structure of the response statistics. Pairwise models have been reported to capture 90% of the correlations, as measured by the multi-information for populations of size *N* = 10 (Schneidman et al., 2006), while our model captures, at most, 70% (Table 1). Yet, pairwise models can also miss important aspects of the collective activity, such as the probability of large population rates (Tkačik et al., 2014), which is captured by our population model.

Both classes of models consider same-time spike patterns, with no regard for the dynamics of spike trains and their temporal correlations. Generalizations of pairwise maximum entropy models to temporal statistics are even harder to solve computationally (Vasquez et al., 2012; Nasser et al., 2013). By contrast, our models of population coupling are fully compatible with any model describing the dynamics of the population rate, such as that by Mora et al. (2015).

## Mathematical derivations

### Derivation of the model form

#### Maximum entropy models

A maximum entropy model is defined by a distribution that maximizes its entropy, as follows:

(13) $$S(P)=-\underset{\sigma }{\sum }P(\sigma )\;\text{log}P(\sigma ),$$

while reproducing a set of chosen statistics. In the case where these statistics are the means of some observables $O_{1}(\sigma ),\ldots ,O_{M}(\sigma )$, the form of the model is given by the following:

(14) $$P(\sigma )=\frac{1}{Z}\exp\left(\overset{M}{\underset{a=1}{\sum }}\mu_{a}\;O_{a}(\sigma )\right),$$

where *Z* is a normalization factor. Equation 14 is obtained by maximizing the entropy while constraining the chosen statistics using the method of Lagrange multipliers. The Lagrange multipliers *μ_a_* are model parameters that must be adjusted so that the mean observables predicted by the model, ${〈O_{a}〉}_{\mu }$ agree with those of the data, ${〈O_{a}〉}_{\text{data}}=(1/n)\overset{n}{\underset{\alpha =1}{\sum }}O(\sigma^{\left(\alpha \right)})$, where $\left(\sigma^{\left(1\right)},\ldots ,\sigma^{\left(n\right)}\right)$ are the *n* activity patterns recorded in the experiment. This fitting procedure is equivalent to maximizing the likelihood of the data under the model $L=\overset{n}{\underset{\alpha =1}{\prod }}P(\sigma^{\left(\alpha \right)})$, assuming that the patterns are independently drawn. The likelihood maximization problem is convex, and the distribution P(σ) maximizing the likelihood is always unique. However, if the constrained observables are linearly related, the optimal set of *μ_a_* is not unique (even though the resulting distribution is), and must be set by choosing a convention.

#### Minimal model

In the minimal model, the statistics we constrain are $P(\sigma_{i}=1)$ for each neuron *i*, and P(K=k) for each *k* = 0,...,*N*. They correspond to the means of the following observables:

(15) $$P(\sigma_{i}=1)=〈\sigma_{i}〉,$$

(16) $$P(K=k)=〈\delta_{K,k}〉,$$

where $\delta_{x,y}$ is Kronecker’s delta, which is equal to 1 if *x* = *y*, and 0 otherwise. Note that while in the main text we use *K* both as a short-hand for $\sum_{i}\sigma_{i}$ and its realization as a random variable, here we distinguish the two by using *K* and *k*, respectively. Applying Equation 14 to this choice of observables $\left(\sigma_{i},\delta_{K,k}\right)$ yields the following:

(17) $$P(\sigma )=\frac{1}{Z}\exp\left(\overset{N}{\underset{k=0}{\sum }}\nu_{k}\delta_{K,k}+\overset{N}{\underset{i=1}{\sum }}\alpha_{i}\sigma_{i}\right)$$

(18) $$=\frac{1}{Z}\exp\left(\nu_{K}+\sum_{i=1}^{N}\alpha_{i}\;\sigma_{i}\right),$$

where each *ν_k_* is associated with the constraint on $〈\delta_{K,k}〉$ and each *α_i_* is associated with the constraint on $〈\sigma_{i}〉$. In the second line, we have used the fact that in the first sum, the only term which is nonzero is the one for which *k* = *K*.

For convenience, we rescale the parameters *ν_K_*, which will give a common form to our three models. We first set *ν_0_* = 0, which is possible because the model is invariant when adding a constant to all *ν_k_* parameters (this changes only the normalization factor *Z*). We then introduce the rescaled parameters *β_K_*, which are defined as *β_0_* = 0 and *β_K_* = *ν_K_*/K for *K* > 0. We have $\nu_{K}=K\beta_{K}=\sum_{i}\sigma_{i}\beta_{K}$, so that the model takes the following form:

(19) $$P(\sigma )=\frac{1}{Z}\exp\left(\overset{N}{\underset{i=1}{\sum }}\left(\alpha_{i}+\beta_{K}\right)\;\sigma_{i}\right).$$

This model has 2*N* − 1 parameters: there are *N* coefficients ${\left(\alpha_{i}\right)}_{i=1}^{N}$ and *N* + 1 coefficients ${\left(\beta_{k}\right)}_{k=0}^{N}$, but *β*_0_ is not used, and the model is invariant under a change in parameters, $\alpha_{i}\prime =\alpha_{i}+c,\;\beta_{k}\prime =\beta_{k}-c$, for any number *c*.

#### Linear-coupling model

The linear-coupling model reproduces $P(\sigma_{i})$ and *P*(*K*), and also the linear correlation between the neuron response $\sigma_{i}$ and the population rate *K*, $〈\sigma_{i}K〉$. The three sets of constrained observables are thus ${\left(\sigma_{i}\right)}_{i=1,\ldots ,N},\;{\left(\delta_{K,k}\right)}_{k=0,\ldots ,N}$, and ${\left(\sigma_{i}K\right)}_{i=1,\ldots ,N}$. With this choice of observables, Equation 14 reads as follows:

(20) $$P(\sigma )=\frac{1}{Z}\exp\left(\overset{N}{\underset{k=0}{\sum }}\nu_{k}\delta_{K,k}+\overset{N}{\underset{i=1}{\sum }}\alpha_{i}\sigma_{i}+\overset{N}{\underset{i=1}{\sum }}\;\gamma_{i}K\sigma_{i}\right)$$

(21) $$=\frac{1}{Z}\exp\left(\nu_{K}+\sum_{i=1}^{N}\left(\alpha_{i}+\gamma_{i}K\right)\;\sigma_{i}\right),$$

where, in addition to the *α_i_* and *ν_k_* parameters, each *γ_i_* parameter is associated with the constraint on $〈\sigma_{i}K〉$. Note that, in general, the inferred parameters *α_i_* and *ν_k_* will be different from the ones inferred in the minimal model. This is due to the fact that the set of observables $\sigma_{i},\;\delta_{K,k}$ and $\sigma_{i}K$ are not independent. Therefore, the parameters *γ_i_* cannot be learned independently from *α_i_* and *ν_k_*.

As for the minimal model, we rescale the parameters *ν_K_* with $\beta_{0}=0$ and $\beta_{K}=\nu_{K}/K$ for *K* > 0, as follows:

(22) $$P(\sigma )=\frac{1}{Z}\exp\left(\overset{N}{\underset{i=1}{\sum }}\left(\alpha_{i}+\beta_{K}+\gamma_{i}K\right)\;\sigma_{i}\right).$$

This model has 3*N* − 2 parameters: there are 2*N* coefficients, ${\left(\alpha_{i}\right)}_{i=1}^{N}$ and ${\left(\gamma_{i}\right)}_{i=1}^{N}$, and *N* + 1 coefficients ${\left(\beta_{k}\right)}_{k=0}^{N}$, but *β*_0_ is not used, and the model is invariant under changes in parameters $\alpha_{i}\prime =\alpha_{i}+c,\;\beta_{k}\prime =\beta_{k}-c+dK,\;\gamma_{i}\prime =\gamma_{i}-d$ for any numbers *c* and *d*.

#### Complete-coupling model

The third maximum entropy model reproduces the joint probability distributions between the response of each neuron and the population rate, $P(\sigma_{i},K)$. The problem reduces to matching $P(\sigma_{i}=1,K)$ for all *i* = 1,…,*N* and *K* = 0,…,*N*, since $P(\sigma_{i}=0,K)$ can be determined through the following:

(23) $$P(\sigma_{i}=0,\;K)=P(K)-P(\sigma_{i}=1,\;K),$$

where the distribution *P*(*K*) is set by the following:

(24) $$\overset{N}{\underset{i=1}{\sum }}P(\sigma_{i}=1,\;K)=KP(K).$$

This holds true because *K* is the number of neurons spiking, so:

(25) $$\overset{N}{\underset{i=1}{\sum }}P(\sigma_{i}=1|K)=\overset{N}{\underset{i=1}{\sum }}〈\sigma_{i}|K〉=〈\overset{N}{\underset{i=1}{\sum }}\sigma_{i}|K〉=K,$$

where we can then multiply both sides by *P*(*K*). Here 〈 . |K〉 stands for the mean conditioned by *K*.

Therefore, we impose that the model reproduces only the statistics $P(\sigma_{i}=1,\;K)$, which are the means of the observables $\sigma_{i}\delta_{K,k}$. Using Equation 14 with this set of observables yields the following:

(26) $$P(\sigma )=\frac{1}{Z}\exp\left(\overset{N}{\underset{i=1}{\sum }}\overset{N}{\underset{k=0}{\sum }}h_{ik}\;\sigma_{i}\delta_{K,k}\right)$$

(27) $$=\frac{1}{Z}\exp\left(\overset{N}{\underset{i=1}{\sum }}h_{iK}\sigma_{i}\right),$$

where each *h_ik_* is associated with the constraint on $〈\sigma_{i}\delta_{K,k}〉$.

This model has N(N−1)+1 parameters: there are N(N+1) coefficients ${\left(h_{iK}\right)}_{i=1,K=0}^{N,N}$, but the *N* coefficients ${\left(h_{i0}\right)}_{i=1}^{N}$ are not used, and only the sum $\sum_{i}h_{iN}$ of the *N* coefficients ${\left(h_{iN}\right)}_{i=1}^{N}$ is used, when all neurons spike simultaneously.

### Regularization

We regularized the empirical population rate distribution *P*(*K*) and conditional firing rates $P(\sigma_{i}|K)$ using pseudocounts. If we denote by $n=2.8\;10^{5}$ the total number of responses *σ* recorded during the experiment and by *n_K_* the number of responses with *K* spikes in the population, the distribution of population rates *K* was computed as follows:

(28) $$P(K)=\frac{n_{K}+\lambda P_{\text{indep}}(K)}{n+\lambda },$$

where $P_{\text{indep}}(K)$ is the distribution of *K* in a model of independent neurons reproducing the empirical firing rates $〈\sigma_{i}〉$. Similarly, if we denote by *n_iK_* the number of responses in which neuron *i* spiked and in which the population rate was *K*, the conditional firing rates were estimated as follows:

(29) $$P(\sigma_{i}=1|K)=\frac{n_{iK}+\lambda P_{\text{indep}}(\sigma_{i}=1|K)}{n_{K}+\lambda },$$

where again $P_{\text{indep}}(\sigma_{i}=1|K)$ is the estimate of the conditional firing rate according to the independent model. The terms scaling as *λ* play the role of pseudocounts. These pseudocounts are not taken to be uniform, but rather follow the prediction of a model of independent neurons. We used *λ* = 1 so that the total weight of pseudocounts is equivalent to a single observed pattern.

### Calculating statistics from the model

We start by providing an analytical expression for the normalization factor, which is defined as follows:

(30) $$Z=\underset{\sigma }{\sum }\exp\left(\overset{N}{\underset{i=1}{\sum }}h_{iK}\sigma_{i}\right).$$

All useful statistics predicted by the model can be derived from the expression of *Z*, as we shall see below. To calculate *Z*, we decompose it as a sum over groups of patterns with the same population activity *K*: $Z=\sum_{k=0}^{N}Z_{k}$ with:

(31) $$Z_{k}=\underset{\begin{array}{l} \sigma \\ K=k \end{array}}{\sum }\text{exp}\left(\overset{N}{\underset{i=1}{\sum }}h_{iK}\sigma_{i}\right)$$

(32) $$=\sum_{i_{1}<...<i_{k}}\text{exp}(\sum_{b=1}^{k}h_{i_{b},k}).$$

We introduce the polynomial $Q(X)=\prod_{i=1}^{N}\left(1+e^{h_{ik}}X\right)$. Expanding *Q*, we can calculate its coefficient of order *X^k^*, denoted by Coeff[*Q*,*X^k^*]. This coefficient is the sum of all the terms having exactly *k* factors $e^{h_{ik}}$, as follows:

(33) $$\text{Coeff}[Q,X^{k}]=\sum_{i_{1}<...<i_{k}}\;\prod_{b=1}^{k}\exp\left(h_{i_{b}k}\right)$$

(34) $$=\sum_{i_{1}<...<i_{k}}\exp\left(\sum_{b=1}^{k}h_{i_{b}k}\right)$$

(35) $$=Z_{k}.$$

It is obtained by recursively computing the coefficients of $\prod_{i=1}^{n}\left(1+e^{h_{ik}}X\right)$, of order up to *X^k^*, for *n* = 1 to *N*, using the following relation:

(36) $$\text{Coeff}[(1+bX)F,X^{l}]=\text{Coeff}[F,X^{l}]+b\;\text{Coeff}[F,X^{l-1}],$$

for any number *b*, polynomial *F*, and order *X^l^*. *Z_k_* can thus be computed in time linear in *kN*, and $Z=\sum_{k}Z_{k}$ can be computed rapidly.

Many statistics of the model can then be calculated by deriving *Z*. For example, the mean observables according to the model in Equation 14 are given by the following:

(37) $${〈O_{a}〉}_{\mu }=\frac{\partial \log Z}{\partial \mu_{a}}$$

This formula gives the following expression for the joint distribution of *σ_i_* and *K*:

(38) $$P(\sigma_{i}=1,\;K)=\frac{\partial \log\;Z}{\partial h_{iK}}$$

(39) $$=\frac{1}{z}Coeff[Xe^{h_{iK}}\underset{j\neq i}{\prod }(1+Xe^{h_{j,K}}),\;X^{K}].$$

Similarly, pairwise correlations are computed using the following formula:

(40) $$〈\sigma_{i}\sigma_{j}〉=\frac{1}{z}\underset{K}{\sum }Coeff[X^{2}e^{h_{iK}+h_{jK}}\underset{j\neq i}{\prod }(1+Xe^{h_{iK}}),\;X^{K}].$$

### Model inference

To learn the model parameters from the data, we maximized the normalized log-likelihood L=(1/n)logL using Newton’s method. The update equation for the parameter values in Newton’s method read as follows:

(41) $$\mu^{\left(t+1\right)}=\mu^{\left(t\right)}-a\;{\text{H}}^{-1}\cdot \nabla L,$$

where *a* is an adjustable step size taken typically between 0.1 and 1. $\mu^{\left(t\right)}={\left(\mu_{a}^{\left(t\right)}\right)}_{a=1,\ldots ,M}$ is the vector of the parameters at iteration *t*; and ∇L and H are the gradient and Hessian of L with respect to the parameters *μ_a_*. In the general context of maximum entropy models (Eq. 14), one can show that the gradient and Hessian read as follows:

(42) $${\left(\nabla L\right)}_{a}=\frac{\partial L}{\partial \mu_{a}}={〈O_{a}〉}_{\text{data}}-{〈O_{a}〉}_{\mu }$$

(43) $$={〈O_{a}〉}_{\text{data}}-\frac{\partial \log Z}{\partial \mu_{a}}$$

(44) $$H_{ab}=\frac{\partial^{2}L}{\partial \mu_{a}\partial \mu_{b}}={〈O_{a}〉}_{\mu }{〈O_{b}〉}_{\mu }-{〈O_{a}O_{b}〉}_{\mu }$$

(45) $$=-\frac{\partial^{2}\log Z}{\partial \mu_{a}\partial \mu_{b}}.$$

where we used Equation 37 for Equation 43 and a similar formula for Equation 45. Both quantities can readily be computed as derivatives of the normalization factor *Z*.

For time efficiency, we only updated the Hessian every 100 iterations of the algorithm. We stopped the algorithm when the fitting error reached 10^−6^. The fitting error was defined as the maximum error on *P*(*K*) and $P(\sigma_{i})$ for the minimal model; on *P*(*K*), $P(\sigma_{i})$ , and $〈K\sigma_{i}〉$ for the linear-coupling model; and on *P*(*K*) and $P(\sigma_{i}|K)$ for the complete-coupling model.

The code for the models inference is available at https://github.com/ChrisGll/MaxEnt_Model_Population_Coupling.

## Synthesis

The decision was a result of the Reviewing Editor Leonard Maler and the peer reviewers coming together and discussing their recommendations until a consensus was reached. A fact-based synthesis statement explaining their decision and outlining what is needed to prepare a revision is listed below. The following reviewers agreed to reveal their identity: Jonathan Pillow, Richard Naud

The reviewers both found merit in this manuscript but still had both conceptual and technical issues.

The discrepancy between the two reviews lies perhaps in their conception of the novelty of model fitting. Is it primarily a technical difference: a likelihood ascent on a likelihood function that is half way between that of a maximum entropy Ising model and pure firing rate?

The authors are encouraged to fully address the points made by both reviewers and respond with a clear point by point response to the reviews that summarizes how they have addressed the remaining issues.

Reviewer 1

The paper describes a novel (to me) method for fitting a particular kind of maximum entropy model that has featured prominently in the recent neural coding literature. It also provides interesting results about the tuning of individual neurons to the population rate ("K"), showing that neurons appear not to be described in terms of a merely linear coupling to K (which would give them monotonic tuning curves in K).

The abstract and intro of the paper are much improved, and I appreciate the authors' efforts to make the paper more accessible to a non-physics audience.

The paper introduces a very interesting method for fitting the population firing rate model to large datasets, and I think this contribution is novel (to me at least) and impressive, and likely to be of use to other researchers who might wish to use this model. However, the paper is very technical and I still worry that it will not be accessible to the readership of eNeuro.

The results presented are quite interesting, but a little bit incomplete. I'm intrigued by the failure of the linear-coupling model to produce single-neuron tuning curves. However, the (relatively) poor performance at predicting pairwise correlations is somewhat dispiriting given that the model actually has more parameters than the Ising model. Two questions I'd like to see addressed more thoroughly: 1) What is the improvement in log-likelihood (or bits) between the linear-coupling and fully-coupled model for predicting spike words? (Apologies if I simply missed this). 2) Can you provide a direct comparison to the Ising (2nd order max-entropy) model? It would be interesting to know if the failure to capture all pairwise correlations or the failure to capture the marginal distribution over K (and coupling from the population to each neuron) is more detrimental to modeling the neural code.

Technical comments:

- The formulation of the model in Equation 2 is still problematic: it makes it appear as though the term in the exponent is linear in the sigma_i's, although it's clearly not because K is a function of the sigma_i's. I appreciate the additional explanation provided in the appendix (equations 17-19), but I still found the equation in the main text mystifying / misleading as written. I'd suggest the authors give the formula involving a sum over \delta_K,k to and adopting different language to indicate that beta_K is itself random here (i.e., not just a parameter), that is, Beta_K is a parameter picked out randomly by the random variable K.

- I'm afraid Equation 24 isn't completely obvious to me. (For K=0 we end up with 0 / 0, so it clearly doesn't work here). Please clarify and provide a derivation or proof or at least pointer to such.

- In the main text it would be helpful to say explicitly how many parameters there are for each model. (If I understand correctly: 2N+1, 3N+1, and N(N+1), respectively).

- "Z_k can thus be computed rapidly using polynomial products" - please provide more detail on how this is carried out (i.e., provide a reference or a name for the method used for rapidly computing these coefficients). This is actually an amazing trick but I'm guessing the average reader (and I include myself) won't know how to actually compute these coefficients. (What is the computational cost? Am I correct in thinking it can be done in N log N time?)

- In Equation 8: D_KL (test || train ) is infinity whenever the test data contains a word not seen in the training data. Are we to assume this never occurred? I have to say the explanation of this method for computing "quality of the model prediction" is not very intuitive or very clearly explained. KL divergence has an interpretation in terms of bits of information about the test data, or in terms of predictive log-likelihood, either of which might help in terms of justification. But in brief, why quantify model fit this way?

Reviewer 2

There is a long tradition of modelling neural systems with methods from statistical mechanics. Hopfield has described how the Ising model used in the study of ferromagnetic materials can be mapped to neural systems and give insights how it memorizes patterns. It was followed by a long string of efforts to assess quantitatively if such pairwise likelihood models can reproduce spiking statistics. The present effort follows in this vein, the authors describe a hybrid approach where the probability of a particular spike pattern in a snapshot of population activity is modeled not as some function of its the N single unit activities only, not as some funciton of its N squared pairwise interaction, but as some function of single unit activities and summed population activity. Using stat. mech. methods and numerical optimization, the authors describe how to infer model parameters and solve for the firing moments. The model is shown to capture firing statistics of the salamander retina. It is argued that the model may be applied to modelling firng statistics of cortical neurons.

The article is technically sound, sufficiently well written for theoretical neuroscientists, and presents novel albeit technical aspects. Yet, I do not see how it addresses interests of the wider neuroscience audience. Assuming that it was submitted in the category 'Novel Tools and Methods', it is hard for me to see that the proposed novel methods will be used by other groups. The article situated its contribution in 1) providing a way to re-analyze the solisters-choristers results of Okun2015, 2) a fast inference for a maximum entropy model, 3) a new maximum entropy model, 4) methods for calculating moments. My point of view is that these are technical contributions of interest to some experts, namely: 1) the re-analyzes is not proposed to yield new results, but a faster convergence. There are many new issues in the cortex that do not appear in the retina (like low pairwise correlations, vast cell numbers and heterogeneous populations), it is not clear if the method applies as is to the cortex. 2) the faster inference is not proven and relies on the not so novel method of numerical gradient ascent. 3) the new entropy model is a nuanced version of the model used by Okun et al. (2015) so it is novel only from the point of view of the maximum entropy model community. And technically, it remains a pairwise model, but with row constraints on the parameters. 4) I agree that the proposed method for calculating moments seems more clever than Okun2015. It seems different from what I have seen from the Bialek's or Tkacik's papers, but I am not sure how novel important the difference is. To be fair, I like the model and the article, I think it would be a nice paper at a more technical venue.
